# Advanced Imaging Modalities for ARIA Detection and Treatment Efficacy Monitoring in Lecanemab Therapy for Alzheimer’s Disease: A Collaborative Prospective Study

**DOI:** 10.1002/alz.095167

**Published:** 2025-01-09

**Authors:** Jude‐Patrick Nnamdi Okafor, Jacob Stefanko, Nayid Jana, Austin A. McCullough, Stephen Jarman, Jessica N Banks, Nelly Joseph‐Mathurin, Edmond Knopp, Tammie L.S. Benzinger

**Affiliations:** ^1^ Washington University School of Medicine, Saint Louis, MO USA; ^2^ Washington University, Saint Louis, MO USA; ^3^ Washington University in St. Louis School of Medicine, St. Louis, MO USA; ^4^ Washington University in St. Louis, St. Louis, MO USA; ^5^ Hyperfine, White Plains, NY USA; ^6^ Hope Center for Neurological Disorders, Washington University in St. Louis, St. Louis, MO USA; ^7^ Mallinckrodt Institute of Radiology, Washington University in St. Louis, St. Louis, MO USA; ^8^ Knight Alzheimer Disease Research Center, St. Louis, MO USA; ^9^ Washington University School of Medicine, St. Louis, MO USA

## Abstract

**Background:**

Alzheimer’s disease (AD) poses a substantial healthcare challenge. Current immunotherapy targeting beta‐amyloid (Aβ) while representing a significant advancement, may be accompanied by potential complications such as amyloid‐related imaging abnormalities (ARIA). ARIA monitoring via high‐field MRI encounters logistical hurdles, exacerbated by regulatory demands for frequent MRI surveillance. This study investigates the integration of a portable ultra‐low field MRI into AD patient monitoring during immunotherapy to optimize ARIA detection, overall workflow and treatment outcomes.

**Method:**

This prospective observational study recruits clinical patients with mild cognitive impairment due to Alzheimer disease underlying evaluation and monitoring for anti‐amyloid monoclonal antibody therapy. Baseline pre‐therapy 3T MRI will serve as a reference for subsequent imaging assessments. Participants undergo Hyperfine Swoop MRI scans at 0.064 tesla alongside their baseline and subsequent sessions, encompassing T1‐weighted, T2‐weighted, FLAIR, and DWI‐ADC sequences.

Monitoring extends for 12 months after the initial clinic visit, enabling real‐time treatment response evaluation. Each participant will undergo ∼5 paired imaging visits, according to the therapy FDA label.

**Result:**

Effectiveness of high‐field and low‐field longitudinal imaging sessions will be compared for detection and evaluation of moderate and severe ARIA‐E (edema). A randomized, blinded assessment approach will be used to evaluate differences in radiologist assessment and treatment plan between field strengths. Patient imaging workflow improvement will be assessed using a survey.

**Conclusion:**

The Hyperfine Swoop MRI, with its portability and accessibility, shows promise as a cost‐effective alternative to the standard 3T MRI with the significant value‐added benefit of optimization of workflow. Further research is needed to confirm these benefits in larger cohorts and assess long‐term reliability. Overall, our study highlights the potential of innovative imaging technologies in improving patient care, monitoring neurodegenerative diseases thereby establishing a more equitable scheme for patient care.

